# Partnerships between an At-Risk Youth CrossFit Program and Local Community Organizations: Focusing on the Antecedents to Partnership Development

**DOI:** 10.3390/sports6030100

**Published:** 2018-09-19

**Authors:** Christina M. Gipson, Natalie Campbell, Nancy L. Malcom

**Affiliations:** 1 Department of Health Sciences and Kinesiology, Georgia Southern University, Statesboro, GA 30460, USA; Nmalcom@georgiasouthern.edu; 2 University of Gloucestershire, Gloucestershire GL50 2RH, UK; ncampbell2@glos.ac.uk

**Keywords:** partnership self-assessment tool-questionnaire, local community partnerships, community sport partnerships, partnership model

## Abstract

A large body of research has established that sport intervention programs can have social, emotional and health benefits for at-risk youth. While research has focused on the positive outcomes associated with these programs, little attention has been given to program inputs. It is recognized that community partnerships can help intervention programs achieve their goals. Yet, how are such partnerships formed and what can help to promote the successful formation of partnerships? This paper provides a detailed account of the partnership implementation process undertaken to develop and deliver a health promotion physical activity program for at-risk youth through the medium of CrossFit in a low socioeconomic area in a rural community in the southeastern United States. Developing successful partnerships serves as a valuable component to help organizations obtain resources and skills needed to initiate and continue programs for underserved populations. The scholars identify and explain how critical success factors such as personal contact, partnership complementarity and fit and the promotion of high levels of commitment and trust, serve as important starting points for developing and maintaining strong community partnerships.

## 1. Introduction

Community based programs attempting to address multidimensional social problems within the youth population can benefit from multiple partnerships across a wide range of organizations [1,2]. Indeed, a significant proportion of youth centered health promotion programs require multiple stakeholder input [3,4]. Such stakeholders often include public health organizations, community sport and physical activity clubs, education providers and local charities. We argue that publications that provide explicit detail about the forming, developing and sustaining of such partnerships in youth development programs is lacking because researchers emphasize program outcomes as opposed program inputs. There is value in publishing information about effective development of partnerships, including discussion of the stakeholders’ involvement in decision making, the allocation of resources and strategies for managing the partnership. Chalip’s [5] call (p. 6), that “we need to discover the characteristics of interventions that are effective or ineffective under particular conditions and pursuant objectives” guided the current paper. The purpose of this manuscript is to provide a detailed account of the partnership development journey of a youth centered health promotion program. The authors apply the Parent and Harvey [6] community based physical activity partnership model while highlighting the fundamental and foundational stages of partnership building that lay the groundwork for successful intervention design and youth development programs.

## 2. Health Inequalities and Sport Intervention Programs: Why Partnerships Are Needed

The promotion of health, both on an individual and community level, is widely recognized as an important component of social progress and societal improvement. However, opportunities and access to programs which support the enhancement of individual and community health vary due to differences in socioeconomic status, race, gender, age and environmental factors [7]. Indeed, the most recent scholarly publications in health inequalities highlight a strong link between socio-economic status and quality of health [8,9,10]. A key challenge for public health organizations in confronting these inequalities is the ability to design, implement, deliver and sustain community based programs that can act upon the social determinants of health. However, as argued by Marmot et al. [11], the success of such programs is often dependent on community insight, the mobilization of resources and a collaborative interest in resolving any locally identified health inequalities. Scholars within the sociology of sport community have responded to the need for health inequality programs to be explored within, between and across populations and have demonstrated the positive contribution that sport and physical activity (PA) can have. Recently, the promotion of health and health benefitting activities through sport and PA programs for youth populations has been shown to contribute to increased social connectedness, reduced engagement in risky behaviors and improved mental health outcomes. Indeed, examples of such studies include the development of healthy self-concept in youth [12]; increased school engagement [13]; increased cognitive function in adolescents [14]; delinquency prevention [15]; improved mental health in youth [16]; and the rehabilitation of young offenders [17]. In light of these findings, youth centered health and social organizations have demonstrated an increased interest in collaborating with organizations in the sport and PA sector to promote subjective wellbeing, increase physical activity, enhance mental health or engage in civic participation in their communities [18,19]. However, as noted by Lucidarme et al. [20], few studies have been conducted on the implementation of effective sport and PA interventions through these collaborative partnerships. Greater emphasis on partnership characteristics, network effectiveness and population challenges is required to increase knowledge at both the theoretical and applied level.

To address the gap in literature, this paper presents a detailed account of the partnership implementation process undertaken to develop and deliver a health promotion physical activity program for ‘at-risk’ youth through the medium of CrossFit in a low socioeconomic area in a rural community in the southeastern United States. To gain an understanding of the partnership developed between the youth CrossFit program and the local community, we opted to use Boutin and Le Cren’s [21] (p. 28) definition of partnership as “an action of sharing ‘good’ and ‘knowledge’ between partners, coupled with a concerted process where the methods of execution and the objectives are known and accepted by all.” This allowed us to have a consistent understanding of partnership development between the youth CrossFit program and the local community.

## 3. Theoretical Model

Parent and Harvey [7] noted that no models or tools had been created to review the development, management and evaluation of partnerships between community-based organizations and physical activity programs. Recognizing this need, they developed a comprehensive model to assess such partnerships based on sport, management and political science domains. The model included several dimensions of partnership and network effectiveness. The model has three main components: antecedents, management and evaluation. The antecedent component addresses the purpose and goals of the program, the general environment, the nature of the partners and partnership development. The management component of the model addresses the attributes of the partnerships by considering whether the partners are a ‘good fit’—based on missions and values of the program and resources. Additionally, in this stage, there is focus on communication and decision making and how partners deal with issues of conflict resolution, power balance, leadership and structure. The last component-evaluation-focuses on evaluating the outcomes of the project and satisfaction with the partnership. Each of the subcomponents can inform partners about the areas where they are satisfied and areas that need work, providing a useful framework on which to scaffold knowledge on the most relevant determinants of effective partnerships. Principally, Parent and Harvey [7] stress the need to apply the model in different circumstances in order to identify the context specific determinants, operations and behaviors of health promotion, sport and physical activity related partnerships. In this study, focus was placed on the antecedent component, the development of partnerships between local community organizations and a CrossFit program for at-risk youth. Such focus enables the reader to understand the complex and intentional thought needed to develop meaningful partnerships.

While the Parent and Harvey [7] model has never before been applied to CrossFit programs for at-risk youth, a small number of sport scholars have published findings on the application of the model to community-based health promotion programs within their geographical spaces. Researchers in Belgium applied the framework to assess the partnership effectiveness of a multi-strategy community based intervention to promote PA in adults (i.e., the ‘10,000 steps’ initiative) [20,22]. These scholars concluded that personal contact, enhanced social skills of staff, aligned political motives and high levels of commitment were factors critical to the success of the network effectiveness and overall delivery of the initiative. Of particular note to our study, the applied work of Bruening, Fuller and Percy [23] provides a multi-level analysis of the campus-community partnership relationships when partnering with nationally recognized charities working with underserved youth to deliver a health promotion program in an area with recognized health inequalities. Bruening et al. [23] stress that the critical success factors of their program lay in the need for reciprocal relationships to be centered on trust at the individual and structural level, frequent interaction and communication between partnership leaders, evidence of commitment to achieving long-term change in the community and manage expectations. Of further interest to us, Marlier et al. [24] applied the Parent and Harvey [7] model to assess capacity building through cross-sector partnerships delivering sports based programs in disadvantaged areas of Belgium. Their application specifically identifies critical factors for success at the practitioner and individual levels. Marlier et al. [24] assert that sustainable and beneficial partnerships are formulated through 8 key determinants: process evaluation, trust, coordination, mutuality, partner complementarity and fit, personal contact, period of collaboration time and policy support. Furthermore, in order to sustain meaningful and beneficial partnerships between public health organizations and non-profit organizations, Beacom and Read [25] argue that partners need to be involved in all aspects of the inception, management and evaluation of the program.

The current research applying the Parent and Harvey [7] model indicates that partnerships with health promotion programs are diverse and complex. Yet, the aforementioned studies provided the authors of this manuscript with exceptional grounding to lay the foundations of a collaborative network when developing partnerships. It was recognized that various types of community organizations need to be involved so each could be effective within the program. Further, the organizations can and should cross sectors while being involved throughout the whole program. The authors conclude that each of these studies highlighted critical success factors that could be considered when setting up partnerships: (i) personal contact; (ii) enhanced social skills of staff; (iii) aligned political motives/partner complementarity and fit; (iv) high levels of commitment; (v) trust; (vi) frequent interaction and communication/coordination; (vii); evidence of commitment to achieving long-term change in the community; (viii) manage expectations; (ix) period of collaboration time; (x) policy support. As we undertook the partnership development journey detailed in this paper, these critical success factors informed each step.

## 4. Developing a Health Promotion Program for at-Risk Youth

The youth development program addressed in this manuscript centers on three important areas of sociological critique: (1) the program will be delivered to at-risk youth; (2) the program will be delivered in a geographical area of low socioeconomic status; and (3) the program will be delivered through a non-traditional physical activity medium. It is important to note that none of the aforementioned studies explored partnership model development within these social parameters. Therefore, attempts to replicate and repeat the processes used by our peers would be impractical and ineffectual; the contextual encompassing of the program is critical. However, we acknowledge a need to be conscious of the contributions of previous studies to ensure every attempt is made to glean best practice insights and embed them within our program formation. Collectively, what we have learned from our peers in applying the Parent and Harvey model [7] to community based physical activity programs is that the factors critical to successful partnership building were not rooted in homogeneous interventions, abundant financial investment or bureaucratic policy. Rather, the programs were successful as a result of genuine human affinity, an authentic purpose of delivery and a shared vision of social improvement. Reflecting on these lessons reminded the authors to not overlook the importance of stakeholder and participant connectedness when establishing a youth CrossFit organization. Therefore, this paper maintains focus on the time taken to develop a youth CrossFit program and the development of strong partnerships.

The local CrossFit program discussed in this paper targeted youth who were deemed ‘at-risk,’ a category often described for individuals in circumstances with: limited social support, inconsistencies in life situations and dependence on school and after-school programs for meals. Haudenhuyse, Theeboom and Nols [26] refer to at-risk youth as being ‘socially vulnerable,’ describing them as having an increased chance of disconnection from social institutions, as well as being targets for negative stigmatization, discrimination, sanctioning and low self-perceptions. Crabbé [27] asserted that the more an individual becomes disconnected, the harder they are to reach. Roberts [28] concurred, suggesting that some individuals seem to drop out of society and fall into the NEET group (not in education, employment or training) suggesting they are not equipped to be active participants helping in society. Therefore, in order to reach this population, intervention programs often offer opportunities that promote and engender positive change by helping youth to discover a new passion, develop employability skills, counter delinquent behaviors, or re-engage with social institutions [28,29].

Over the past two decades there has been a growth in research to understand interventions for at-risk youth that use sport and/or PA as the vehicle for personal betterment [30,31,32,33,34]. These sports-based programs range from “crime prevention and public health to daycare, juvenile delinquency and teenage pregnancy to gangs, drugs and violence education” [31] (p. 339) and often seek to recruit poor, disadvantaged and minority youth. Studies of the sport-based interventions have been identified as ‘social problem prevention’ programs and are often recognized separately to general health promotion programs, despite improved physical health being a byproduct of such initiatives [35]. Studies have shown to deliver positive outcomes through such programs that are for youth who have limited material and social resources, limited access to physical activity, lower academic achievement and greater incidences of health-related problems such as obesity and depression [36]. Furthermore, many social problem prevention sport and PA interventions aim to support and promote the development of qualities often deemed to be lacking in at-risk youth [37]. These include programs that purposefully cultivate interpersonal skills, quality relationships, emotion management, problem-solving, cognitive competencies, self-efficacy, commitment to schooling and academic achievement.

### CrossFit Programs for at-Risk Youth

The fitness regime of CrossFit has seen an increased societal interest as a space for physical and personal change. The first CrossFit box (“box” is CrossFit terminology used for gym) opened in 2000 and now there are more than 13,000 affiliates worldwide [38]. CrossFit offers functional workouts that simultaneously use cardiovascular endurance, strength and power and gymnastics movements. Additionally, CrossFit offers a sense of community, friendships and camaraderie [39]. CrossFit Kids (CFK) programs were developed in 2004 to provide organized training methods designed for people under the age of 18 [40]. CFK aims to develop the foundational principles of physical literacy within an environment where individual success and progression is not dependent on, nor measured against, peers [41]. A principle training methodology of CrossFit is that all sessions can be scaled for each participant, meaning weights and movements are adjusted to fit the participant’s abilities. Therefore, offering CrossFit to youth who have various experiences with sport and physical activity can encourage participants to focus their attention, effort and valuation on their own abilities and achievements at each session. Scholars have found success with offering CrossFit in middle and high school [40,41,42,43] and afterschool settings [44,45]. 

CrossFit also has a history of being offered to underserved youth populations. Steve’s Club was the first official organization to develop CrossFit programs for inner-city youth at-risk in Camden, NJ in 2008 [4,5]. As of 2016, Steve’s Club has helped 34 other programs targeting underserved populations complete the necessary procedures to become non-profit 501(c)(3) organizations [4,5]. Additionally, based on a Google search, social media and community forums, there are estimated to be more than 100 CrossFit programs for youth at-risk across the United States; some are linked to Steve’s Club (e.g., Steve’s Club Denver, Steve’s Club Agoge and Steve’s Club Washington, D.C.) and others are not (e.g., Ryan’s Club, TFR Foundation, Tomorrow Luminaries and Performance Initiatives). Programs are run out of CrossFit boxes, in parks and even in school settings. Common values stated in each program include integrity, respect, teamwork, support, positivity, discipline, character and community. While all of these programs cater to underserved youth, the organizers define ‘underserved youth’ according to their own community standards and their demographics.

CrossFit programs for underserved youth are run at the community level. Often the programs themselves must find ways to generate funding to cover the costs of membership, clothing, equipment and in many cases food and transportation [46]. For example, Steve’s Club uses a multi-pronged approach to address the challenge of fundraising. In addition to hosting an annual fundraising event called Beat the Streets, the club asks for donations of fitness clothing, equipment, money and talent, while also seeking out sponsorships and scholarships [46]. Parent and Harvey [47] recognized that community level sport programs are typically run solely by the organizations, though more recent trends have seen the establishment of partnerships. Developing partnerships is a valuable component to help organizations obtain resources and skills to continue programs for underserved populations. Community organizations, like CrossFit programs for underserved youth, can set-up partnerships with private, public, commercial and other community organizations [8,48,49,50,51,52]. Through these relationships, community partners share and agree on objectives and activities [7]. Babiak and Thibault [9] underscored the importance of collaboration, because it provides access to resources, expertise, knowledge, structure and programs.

## 5. The Preliminaries of Partnership Formation

There are major challenges in developing, managing and maintaining organizations aiming to provide physical activity for underserved youth. In this paper, the authors focus on the antecedent component of Parent and Harvey’s [7] model to explain how the partnerships were developed with the youth CrossFit program. Kidd [53] reminds us that grassroots organizations tend to be “woefully underfunded, completely unregulated, poorly planned and coordinated and largely isolated from mainstream development efforts” (p. 376). Because funding is typically recognized as the most important recurring issue for grassroots organizations, the organizers of the youth CrossFit program sought different funding sources that could assist with the program development and maintenance. Initial focus was on securing grant money, which the organization was able to achieve through a national foundation for at-risk youth. The purpose of the grant was to pay for clothing, equipment and qualified coaches.

Additionally, Schulenkorf, Sugden and Burdsey [54] assert that organizations need to develop a clear and concise understanding of the appropriate structures and processes needed to develop, manage and maintain programs and interventions for underserved youth. Therefore, a committee was created to serve as a review board. This review board included five members representing two people directly involved in the organization and three who had been previously uninvolved. The role of the committee was to help regulate the program and review the future direction. Early in the process, much of the committee’s attention was focused on identifying potential partners that could assist with areas where the organization could not act alone. Researchers Svensson and Hambrick [55] highlight the significance of external partnerships to provide necessary support such as access to sport facilities, financial support (in forms of discounted prices and in-kind donations) and human resources. According to MacIntosh and Spence [50], having well thought out partnerships can hugely impact program design, delivery and outcomes. Through the youth CrossFit program, the organizers wanted to show: (a) the ability to secure funding, (b) a group tasked to review regulations and (c) that considerations were given to develop significant partnerships before entering the local community. These steps were taken both to lay a solid foundation for the intervention program and to signal to the community and to potential partners that the program was meant to be long term and stable. Because of this careful planning and attention to the antecedents of partnership development, more time was given to outlining the partnership mission statement and partnership agreement.

In addition to issues of funding and organizational planning, the community context is also important and needs to be taken into consideration. Organizers of the youth CrossFit program analyzed the local county to gain a clear understanding about the environment. A number of contextual factors arose that could not be addressed without external support, further underscoring the importance of developing partnerships in order to create a successful intervention program. Some of the main obstacles facing the local community centered on high levels of poverty, low education levels, lack of transportation and few available fitness programs for youth. In 2015 where the program was being held, the county-wide per capita annual income was $18,719. Only 29% of adults had a bachelor’s degree or higher. There was no public transportation in the form of buses, trains, or city-wide walkways in the community. This means that if youth want to go somewhere, they have to walk on roads, arrange a personal ride, or simply not go. The organizers realized in order to reach underserved youth, they would have to go to the youth to run a program or find some form of transportation. Finally, there were no fitness programs for underserved youth beyond the county recreation department and school activities. Therefore, bringing in a new fitness routine, like CrossFit, provided a non-traditional, individualized program that represented a significant departure from what was available in the area. Because of limited knowledge about the activity, it was important to hire well qualified, experienced CrossFit staff in order for the intervention to have the best chance at success.

## 6. Developing Partnerships with the Local Community

Considering Babiak and Thibault’s [9] concern that sport leaders and managers often “lack the skills to identify, establish and manage these partnerships effectively” (p. 118), the organizers of the youth CrossFit program took a number of steps before reaching out to the community. Securing grant funding was important to the organizers in hopes that the grant would signal to potential partners their high level of commitment to the program. The one-year grant from the national foundation for at-risk youth, which led to the development of a non-profit 501(c)(3), was highly publicized in the local paper, on the local university’s website and placed in partnership letters. The organizers also felt that having the board comprised both of people involved in the programmatic part of the organization and people not directly involved would show potential partners that the organization had a structure conducive to oversight and regulation. During partnership meetings, the organizers explained the purpose of the committee and emphasized the role that the committee would play in offering longer-term stability to the intervention program.

The committee met to discuss the rural community and identified the major contextual factors that needed to be addressed so the organization could be successful and meet the goals. Realizing that local obstacles included the lack of public transportation, a high percentage of low income population and the need for properly trained staff, the committee recommended partners and sponsors from the local community that could help address these shortcomings. The organizers set-up meetings with community organizations who could assist with specific needs for the program. During the meeting, information was given about the program, how the community organization could be useful and how the program could benefit the organization. Informational sheets were given to the individual who would serve as the point of contact. Follow-up meetings were scheduled with the interested organizations where the point of contact and program leaders discussed the responsibilities of both parties.

The youth CrossFit program established four significant partnerships to address the community’s shortcomings. All four partnerships have been maintained for two complete years and each partnership renewed with the program for the third year. A brief description of the organizations and partnership relationships are provided.

- A national afterschool organization. For the first year of operation, all members of the youth CrossFit program came from the local county location of the national afterschool organization. As the partnership was developed, the local county location of the national afterschool organization described the members as being at-risk. According to the administrators, 94% of their membership come from minority and/or low-income families in the rural community. Many come from single-parent or caregiver homes and the children have limited options for places to go other than their home, school and the afterschool location. Additionally, many of the participants depend on Federal Food Assistance Programs and meals provided from the afterschool organization throughout the academic year. The local county location of the national afterschool organization received a federal grant to fund the organization transporting their members to activities that are not provided on site. The local county location of the national afterschool organization formally agreed to: (1) recruit middle school aged members and transport them to the local CrossFit facility (10-mile round trip); (2) provide an adult supervisor from their organization; (3) provide shoes for each participant which would be stored at their facility; and (4) have food available for each child when they return from CrossFit.
- Local CrossFit. The local CrossFit facility, established in 2012, started a CrossFit Kids program in 2014. In 2015, the business was doing well and the owner was interested in getting more involved in the community. Coaches who held the CrossFit Level 1 and CrossFit Kids certificates were recruited from the local facility. Additionally, two of the coaches had experience working in the public school system. The local CrossFit box was excited to set-up a partnership where they formally agreed to: (1) assist with the structure and design of the program; (2) provide a qualified coach; (3) provide space and equipment for classes; (4) include the program on the insurance policy held by the facility; and (5) provide discounted memberships to the youth members.
- Local Pediatric office. There are ten pediatric businesses in the 10-mile radius that spans this community. As the youth CrossFit program was being established, one local pediatric office showed interest in partnering with the youth CrossFit program. This pediatric office was a good fit because their organization already served the local community. The medical organization described working with patients in the same population (e.g., patients whose families require federal food and housing assistance, live in single-parent homes and live in low-income environments). The partnership was important because the organization agreed to provide a large financial donation. Additionally, free physicals were provided to each participant between 5–8 p.m., which was valuable because the pediatric office hours of operation are weekdays between 8 a.m. and 5 p.m. Organizers discovered that one reason many at-risk children do not participate in school sports was due to the lack of flexibility in medical office hours, resulting in youth not being able to complete the physical exams that are required in order to participate. These school-based sporting activities often required parents to leave work in order to facilitate their children’s participation in the activities. By providing free physicals later in the evening, parents/caretakers not only did not have to worry about insurance coverage or out-of-pocket charges but the flexible hours allowed parents/caretakers to complete the paperwork and be present for the physical exam after their work day had ended. The pediatric office also agreed to be available for any program participant who needed medical attention as a result of participation in the program (note, none of the youth participants have needed medical intervention over the three years of the program’s operation).
- A local, youth-focused private foundation. The youth-focused foundation was created to invest in the lives of young people. The foundation aims to reduce the number of barriers that at-risk youth experience and to expose them to new and different opportunities in the world. The youth-focused foundation formally agreed to provide financial backing that goes towards: (1) paying memberships during the academic year and one scholarship for a participant to attend a summer camp; and (2) provide shoes, shorts and under clothes for any participant so each person can have access, opportunities and new experiences.

## 7. Discussion about Critical Success Factors

Throughout this process of partnership development, we sought to rely on findings from previous research to enhance the proposed intervention program and boost its chances of succeeding. The development of the partnership among the four entities described above was guided by our review of the collective critical success factors identified in literature utilizing the Parent and Harvey [7] model for community-based partnerships for health intervention programs. In alignment with previous literature, we found that the most important factors for laying the groundwork for our CrossFit for at-risk youth intervention program were: (1) personal contact; (2) partner complementarity and fit; and (3) promoting commitment and trust among partners. In our development, we found overlap with promoting commitment and trusting partners and therefore, these two factors were merged and discussed together.

**Personal contact**. One person from each organization was identified as the contact person. This individual was expected to act as the liaison between the partner organization and the program. This person was responsible for funneling information to their stakeholders and making decisions for the partnership. Identifying and cultivating this personal contact within each partner organization during the planning stages, prior to the implementation of the intervention program, was critical for the success of the overall partnership. Having this personal contact in place benefited the partnership in three specific ways: (1) reducing confusion and the potential for mixed messages; (2) enhancing the efficiency of communication and the speed with which decision-making occurred; and (3) assisting in maintaining the partnership’s mission and goals.

An outcome from identifying the personal contact was the development of high levels of communication. Since this person was responsible for identifying the best way to work through the partnership, the personal contact was responsible for guiding how decisions would be made for their organization and for recommending how best to address any issues that arose in the partnership. By maintaining open communication with the contact person, we were able to coordinate programmatic needs and plans with all of the community partners in order to streamline program management.

**Partnership complementarity and fit**. Branding and public image were important considerations for our program as well as for each of the partners involved. Therefore, we sought to identify potential partner organizations that had positive public images and had similar missions. For example, each of the organizations we contacted about entering into a partnership worked with youth and all four partners had a vested interest in providing health opportunities for youth. Therefore, the partners were able to work together, each identifying resources that they were able to provide to support the overall partnership and the program.

**Promoting high levels of commitment and trust**. Even before contacting potential partners, the organizers of the program made intentional efforts to develop a strong foundation to show their commitment to the program. For example, the advisory board was in place and the initial grant was secured before any contact was made with potential community partners. The organizers wished to demonstrate their serious intentions with the project, which was also a sign to partners that the program was poised for longer-term success. Early discussions with potential partners emphasized the careful planning that had already taken place to structure the program and new partners were brought into the discussion so that their voices and viewpoints were valued as programmatic planning continued to unfold. By encouraging partner organizations to be involved and have a voice with various decisions from the beginning, the organizers found that both trust and increased commitment were being built. After the program was implemented, community partners continued to be closely involved. They were encouraged to attend CrossFit sessions with the youth throughout the program and representatives of two organizations have come to the gym to join to participate in the activities. The partners were also able to follow the progress of the youth through biannual newsletters.

Kidd [53] highlighted that non-profit and programs for youth at-risk were underfunded. Therefore, organizers of the youth CrossFit program that served as the focal point of this paper recognized that partnerships would be needed if the program were to succeed. Rather than rushing headfirst into arranging partnerships, however, organizers took the time to plan. Organizers became familiar with prior research on sustainability of partnerships in youth development programs [20,22,23,24]. The critical success factors that were most useful for this project informed the plans of the four partnerships presented in this paper. Before reaching out to potential partners, organizers first chose to secure external funding for the program which showed the organizational commitment [23]. Particularly, if an intervention program is located in a poverty-stricken area, funding can be more difficult to attain and therefore, partnerships with various organizations for financial support are often necessary. In addition to securing funding, organizers defined the structure of the youth CrossFit program by creating a review board that would oversee the program. Having this organizational structure already in place was yet another signal to potential community partners that the intervention program was poised for success and that partnering with the youth CrossFit program would be worthwhile. Even when organizers make careful plans to implement their program, the development of community partnerships does not always go smoothly. In the case discussed here, partnership development was met with two main challenges. First, the organizers were intentional with the targeted community partners because of the value of the brand. Both the local CrossFit box and the local county afterschool program are affiliated with well-known national organizations that have carefully cultivated their images and that recognize a need to protect these images. As this intervention program was still in the planning stages, there was an opportunity to partner with a large, local organization that had a large amount of resources which could benefit the youth program. However, they were linked to alcohol and social settings for young adults. The organizers were concerned that partnering with this entity could negatively affect the image of the local youth program, as well as damage the national brands of both CrossFit and the national afterschool program. Second, some of the partnerships were slow to develop because of schedule conflicts with the organizers and the personal contacts. Although both parties were interested and wanted to move forward, some decisions and actions took slightly longer than anticipated. The youth intervention program described here could have been implemented much more quickly had we paid less attention to the antecedents of partnership development. However, we believe that the time and attention devoted to planning pays off in long-term stability and success of the program. 

## 8. Conclusions

This paper highlights the importance of developing intentional partnerships when creating a sport intervention program for at-risk kids in low-income and underserved communities. Sport intervention programs are widely recognized as an effective vehicle for improving children’s health and well-being. However, scholarly research on intervention programs tend to focus on program outcomes and neglect the value of sharing the processes of establishing a successful, long-term organization. We argue that in order for intervention programs to succeed, careful attention must be paid to the establishment of the organization. Furthermore, we recognize that when serving at-risk kids in underserved communities, successful sport intervention programs often rely on the development of partnerships, which adds a layer of complexity to the planning and development process.

Recognizing the need for a tool to manage and evaluate local partnerships for community-based youth sport and physical activity programs, Parent and Harvey [7] proposed a comprehensive model to assess such programs. The Parent and Harvey model includes three main components: antecedents, management and evaluation. In this paper, we applied the Parent and Harvey model to a youth CrossFit program. We focus solely on the antecedents of partnership development, taking a close look at the purpose and goal of the program, the general environment of the local community and partnership planning. In future work, we will apply the Parent and Harvey partnership assessment model [7] to both the management and evaluation of the youth CrossFit program. 

At the conclusion of our planning process, the youth CrossFit program entered into four significant community partnerships, which ultimately led to the implementation of a successful sport intervention program for at-risk kids in our local community. Organizers of youth development programs in other communities can learn from the example of the youth CrossFit program discussed here. While there is ample evidence that sport intervention programs can have positive outcomes, there is much less attention given to the strategic planning that is required in order to develop partnerships that lead to more effective intervention programs. In sharing the experiences of the local youth CrossFit program, we provide a roadmap for other organizers of youth development programs. Organizers who are cognizant of the antecedents of partnership development will better understand the importance of planning and can take steps that lead to even stronger community partnerships that enhance the overall success of youth development programs.

## References

[1] Alexander J.A., Comfort M.E., Weiner B.J., Bogue R. (2001). Leadership in Collaborative Community Health Partnerships. Nonprofit Manag. Leadersh..

[2] Babiak K., Thibault L. (2009). Challenges in Multiple Cross-Sector Partnerships. Nonprofit Volunt. Sect. Q..

[3] Diamond J. (2001). Managing Change or Coping With Conflict?—Mapping The Experience Of A Local Regeneration Partnership. Local Econ..

[4] Lindsey I. (2013). Community collaboration in development work with young people: Perspectives from Zambian communities. Dev. Pract..

[5] Chalip L. (2006). Toward a Distinctive Sport Management Discipline. J. Sport Manag..

[6] Parent M.M., Harvey J. (2009). Towards a Management Model for Sport and Physical Activity Community-based Partnerships. Eur. Sport Manag. Q..

[7] Fraser-Thomas J., Côté J., Deakin J. (2005). Youth sport programs: An avenue to foster positive youth development. Phys. Educ. Sport Pedagog..

[8] King N.B., Harper S., Young M.E. (2012). Use of relative and absolute effect measures in reporting health inequalities: Structured review. BMJ.

[9] Uphoff E.P., Pickett K.E., Cabieses B., Small N., Wright J. (2013). A systematic review of the relationships between social capital and socioeconomic inequalities in health: A contribution to understanding the psychosocial pathway of health inequalities. Int. J. Equity Health.

[10] Thomson K., Hillier-Brown F., Todd A., McNamara C., Huijts T., Bambra C. (2018). The effects of public health policies on health inequalities in high-income countries: An umbrella review. BMC Public Health.

[11] Marmot M., Friel S., Bell R., Tanja A.J.H., Taylor S. (2008). Closing the gap in a generation: Health equity through action on the social determinants of health. Lancet.

[12] Babic M., Morgan P., Plotnikoff R., Lubans D., Lonsdale C., White R. (2014). Physical activity and physical self-concept in youth: Systematic review and meta-analysis. J. Sci. Med. Sport.

[13] Owen K.B., Parker P.D., Van Zanden B., MacMillan F., Astell-Burt T., Lonsdale C. (2016). Physical Activity and School Engagement in Youth: A Systematic Review and Meta-Analysis. Educ. Psychol..

[14] de Greeff J.W., Bosker R.J., Oosterlaan J., Visscher C., Hartman E. (2018). Effects of physical activity on executive functions, attention and academic performance in preadolescent children: A meta-analysis. J. Sci. Med. Sport.

[15] Taheri S.A., Welsh B.C. (2016). After-School Programs for Delinquency Prevention: A Systematic Review and Meta-Analysis. Youth Violence Juv. Justice.

[16] Lubans D., Richards J., Hillman C., Faulkner G., Beauchamp M., Nilsson M., Kelly P., Smith J., Raine L., Biddle S. (2016). Physical Activity for Cognitive and Mental Health in Youth: A Systematic Review of Mechanisms. Pediatrics.

[17] Woods D., Breslin G., Hassan D. (2017). A systematic review of the impact of sport-based interventions on the psychological well-being of people in prison. Ment. Health Phys. Act..

[18] Casey M.M., Payne W.R., Eime R.M., Brown S.J. (2009). Sustaining health promotion programs within sport and recreation organisations. J. Sci. Med. Sport.

[19] Theeboom M., Haudenhuyse R., De Knop P. (2010). Community sports development for socially deprived groups: A wider role for the commercial sports sector? A look at the Flemish situation. Sport Soc..

[20] Lucidarme S., Cardon G., Willem A. (2016). A Comparative Study of Health Promotion Networks: Configurations of determinants for network effectiveness. Public Manag. Rev..

[21] Boutin G., Le Cren F. (2004). The Partnership: Between Utopia and Reality.

[22] Lucidarme S., Marlier M., Cardon G., De Bourdeaudhuij I., Willem A. (2013). Critical success factors for physical activity promotion through community partnerships. Int. J. Public Health.

[23] Bruening J.E., Fuller R.D., Percy V.E. (2015). A Multilevel Analysis of a Campus-Community Partnership. J. Serv. Learn. High. Educ..

[24] Marlier M., Lucidarme S., Cardon G., De Bourdeaudhuij I., Babiak K., Willem A. (2015). Capacity building through cross-sector partnerships: A multiple case study of a sport program in disadvantaged communities in Belgium. BMC Public Health.

[25] Beacom A., Read L., Houlihan B., Green M. (2011). Right to Play: Sustaining Development Through Sport. Routledge Handbook of Sport Development.

[26] Haudenhuyse R., Theeboom M., Nols Z. (2012). Sports-based interventions for socially vulnerable youth: Towards well-defined interventions with easy-to-follow outcomes?. Int. Rev. Sociol. Sport.

[27] Crabbe T. (2007). Reaching the ‘hard to reach’: Engagement, relationship building and social. Int. J. Sport Manag. Mark..

[28] Roberts S. (2011). Beyond ‘NEET’ and ‘tidy’ pathways: Considering the ‘missing middle’ of youth transition studies. J. Youth Stud..

[29] Andrews J.P., Andrews G.J. (2003). Life in a secure unit: The rehabilitation of young people through the use of sport. Soc. Sci. Med..

[30] Coalter F. (2013). ‘There is loads of relationships here’: Developing a programme theory for sport-for-change programmes. Int. Rev. Sociol. Sport.

[31] Hartmann D. (2001). Notes on Midnight Basketball and the Cultural Politics of Recreation, Race, and At-Risk Urban Youth. J. Sport Soc. Issues.

[32] Lawson H.A. (2005). Empowering people, facilitating community development, and contributing to sustainable development: The social work of sport, exercise, and physical education programs. Sport Educ. Soc..

[33] Schulenkorf N. (2010). The roles and responsibilities of a change agent in sport event development projects. Sport Manag. Rev..

[34] Vail S.E. (2007). Community Development and Sport Participation. J. Sport Manag..

[35] Goodman E., Slap G.B., Huang B. (2003). The Public Health Impact of Socioeconomic Status on Adolescent Depression and Obesity. Am. J. Public Health.

[36] Hartmann D., Wheelock D. (2002). Sport as prevention? Minneapolis’ experiment with late night basketball. CURA Rep..

[37] Catalano R.F., Berglund M.L., Ryan J.A., Lonczak H.S., Hawkins J.D. (2004). Positive Youth Development in the United States: Research Findings on Evaluations of Positive Youth Development Programs. Ann. Am. Acad. Polit. Soc. Sci..

[38] Bailey B., Benson A.J., Bruner M.W. (2017). Investigating the organisational culture of CrossFit. Int. J. Sport Exerc. Psychol..

[39] Davies M.J., Coleman L., Babkes Stellino M. Why CrossFit?: Participants’ Basic Psychological Needs and Motives. Presented at the 2014 North American Society for Sports Management Conference.

[40] Moran K. (2014). The Effects of Using the CrossFit Kids Program on Academics and Fitness. Ph.D. Dissertation.

[41] Sibley B.A. (2012). Combining contemporary with classic: CrossFit in sport education. JOPERD.

[42] Eather N., Morgan P.J., Lubans D.R. (2016). Improving health-related fitness in adolescents: The CrossFit Teens™ randomised controlled trial. J. Sports Sci..

[43] Sánchez-Alcaraz Martínez B.J., Gómez-Mármol A. (2015). Perception of effort, enjoyment and learning in secondary students in physical education lessons during a CrossFit teaching unit. Sport TK Rev. EuroAmericana De Cienc. Del Deporte.

[44] Kozub F. (2013). Using the snatch and CrossFit principles to facilitate fitness. J. Phys. Educ. Recreat. Dance.

[45] Gipson C.M., Wilson C.H., Burdette T. (2016). Lessons from youth perceptions of a CrossFit after-school program. GAHPERD.

[46] Steve’s Club National Program 2016 Annual Report. http://www.stevesclub.org/annual_report/.

[47] Parent M.M., Harvey J. (2017). A partnership-based evaluation of a community-based youth sport and physical activity programme. Sport Soc..

[48] Bowers M.T., Green B.C. (2013). Reconstructing the Community-Based Youth Sport Experience: How Children Derive Meaning from Unstructured and Organized Settings. J. Sport Manag..

[49] Lindsey I. (2006). Local Partnerships in the United Kingdom for the New Opportunities for PE and Sport Programme: A Policy Network Analysis. Eur. Sport Manag. Q..

[50] MacIntosh E., Spence K. (2015). Management challenges in delivering an international sport and development program. Sport Bus. Manag. Int. J..

[51] Phillpots L. (2013). An analysis of the policy process for physical education and school sport: The rise and demise of school sport partnerships. Int. J. Sport Policy Polit..

[52] Bablak K. (2007). Determinants of interorganizational relationships: The case of a Canadian nonprofit sport organization. J. Sport Manag..

[53] Kidd B. (2008). A new social movement: Sport for development and peace. Sport Soc..

[54] Schulenkorf N., Sugden J., Burdsey D. (2014). Sport for development and peace as contested terrain: Place, community, ownership. Int. J. Sport Policy Polit..

[55] Svensson P.G., Levine J. (2017). Rethinking Sport for Development and Peace: The Capability Approach. Sport Soc..

